# Associations of eicosapentaenoic acid and docosahexaenoic acid intakes with cardiovascular and all-cause mortality in patients with diabetes: Result from National Health and Nutrition Examination Survey 1999–2008

**DOI:** 10.3389/fcvm.2022.1031168

**Published:** 2023-01-09

**Authors:** Nian Huang, Fang Wang, Shiyang Li, Xiaobing Zhai, Wenzhi Ma, Keyang Liu, Haytham A. Sheerah, Jinhong Cao, Ehab S. Eshak

**Affiliations:** ^1^School of Public Health, Wuhan University, Wuhan, China; ^2^Department of Biostatistics, School of Public Health, Xuzhou Medical University, Xuzhou, Jiangsu, China; ^3^Center for Medical Statistics and Data Analysis, Xuzhou Medical University, Xuzhou, Jiangsu, China; ^4^Faculty of Applied Sciences, Center for Artificial Intelligence Driven Drug Discovery, Macao Polytechnic University, Macao, Macau SAR, China; ^5^Public Health, Department of Social Medicine, Graduate School of Medicine, Osaka University, Osaka, Japan; ^6^Health Promotion and Health Education Research Chair, King Saud University, Riyadh, Saudi Arabia; ^7^Health Promotion Center, Riyadh, Saudi Arabia; ^8^School of Management, Hubei University of Chinese Medicine, Wuhan, China; ^9^Research Center for the Development of Traditional Chinese Medicine, Hubei Province Project of Key Research Institute of Humanities and Social Sciences at Universities, Wuhan, China; ^10^Public Health and Community Medicine, Faculty of Medicine, Minia University, Minya, Egypt; ^11^Advanced Clinical Epidemiology, Medical Data Science Unit, Public Health, Graduate School of Medicine, Osaka University, Osaka, Japan; ^12^Public Health, School of Health, Calvin University, Grand Rapids, MI, United States

**Keywords:** EPA, DHA, CHD, CVD, diabetes

## Abstract

**Introduction:**

The evidence on eicosapentaenoic acid (EPA) and docosahexaenoic acid (DHA) intake status and long-term mortality among people with diabetes is scarce. This study aimed to investigate the relationship between EPA and DHA intakes with all-cause and cause-specific mortality in adults with diabetes.

**Methods:**

This study included 2,991 adults with diabetes from the National Health and Nutrition Examination Survey (NHANES) 1999–2008. Death outcomes were ascertained by linkage to the database records through 31 December 2015. Cox proportional hazards regression models were used to estimate hazard ratios (HRs) and 95% confidence intervals (CIs) for mortality from all causes, cardiovascular disease (CVD), and coronary heart disease (CHD) in patients with diabetes.

**Results:**

Among 2,991 patients with diabetes, the mean age was 61.9 years (55.2% males). During the mean follow-up duration of 9.4 years, a total of 1,091 deaths were documented, of which 273 were due to CVD, including 227 CHD deaths. EPA and DHA intakes were associated with lower mortality risks, especially that of CVD. After adjusting for demographic, major lifestyle factors, overall dietary intake patterns, and history of hypertension and dyslipidemia, the multivariable HRs (95% CIs) of mortality risk comparing Q4 to Q1 of EPA intake were 0.55 (0.33–0.92; *P*-trend = 0.019) for CHD, 0.55 (0.36–0.83; *P*-trend = 0.005) for CVD, and 0.91 (0.70–1.18; *P*-trend = 0.264) for all-cause. The respective HRs (95% CIs) comparing Q4 to Q1 of DHA were 0.60 (0.37–0.98; *P*-trend = 0.051) for CHD, 0.58 (0.38–0.89; *P*-trend = 0.014) for CVD, and 0.92 (0.72–1.18; *P*-trend = 0.481) for all-cause. In subgroup analysis, we found that the association trends of EPA and DHA intakes with death risk remained robust among patients with diabetes, especially among those who are old, female, those with higher BMI, and dyslipidemia patients with CVD and CHD.

**Discussion:**

In the USA, higher EPA and DHA intakes were associated with a lower risk of CHD and CVD mortality in patients with diabetes. Our study supports the benefits of adequate EPA and DHA intakes in promoting the health of patients with diabetes.

## 1. Introduction

As a clear global public health problem, the incidence rate of type 2 diabetes mellitus (T2DM) has risen sharply in the past few decades. According to the Global Burden of Disease Study (GBD) 2019, the prevalence of T2DM worldwide has reached 5.6%, and more than 450 million people are affected by this disease (1). In the USA, the prevalent cases in 2019 were over 38 million (2), and the number of deaths from T2DM in 2020 was 101,106, with an increase of 27% from 79,535 in 2005 (3).

Some studies have shown that diabetes is associated with a higher risk of death from many diseases, especially cardiovascular disease (CVD) (4). Diabetes is an important risk factor for coronary heart disease (CHD), stroke, and peripheral vascular disease (PAD) (5), and can increase about double the risk of CHD and stroke mortality (6).

Results from prospective cohort studies and randomized controlled trials have demonstrated the protective effect of *n*-3 fatty acids on the cardiovascular system (7–10). Eicosapentaenoic acid (EPA) and docosahexaenoic acid (DHA) are *n*-3 long-chain polyunsaturated fatty acids (LC-PUFAs) ubiquitous in marine animals and plant plankton and have effective protection for blood vessels (11). Notably, consuming seafood one or two times a week could protect against CVD and reduce mortality risk due to the high content of DHA and EPA (12). These health benefits of LC-PUFAs may alleviate physiological conditions, including hyperlipidemia, diabetes, cancers, inflammation, and neurodegenerative diseases (13–16). Some clinical trials have evaluated the impact of LC-PUFAs supplementation on the risk of CVD events. Still, the results were inconsistent and varied due to the characteristics of the studied sample. A study of cardiovascular events in diabetes (17) (*n* = 15,480) found no reduction in CVD risk. The Vitamin D and Omega-3 Trial (VITAL) (18) (*n* = 25,871) found a non-statistically significant 7% reduction in the risk of CVD events and an unexpectedly high 28% reduction in the risk of myocardial infarction (MI), a pre-specified secondary outcome. On the contrary, the Reduction of Cardiovascular Events with Icosapent Ethyl-Intervention Trial (REDUCE-IT) (19) (*n* = 8179) that studied the effect of Vascepa (icosapent ethyl), a highly concentrated ethyl ester form of EPA on patients with mostly borderline and mildly high triglycerides who were taking statins, found a statistically significant 25% reduction in the risk of CVD events.

As CVD mortality in patients with diabetes is a common public health and clinical issue, and the evidence of LC-PUFAS on the cardiovascular health of patients with diabetes is still limited, therefore, the purpose of this study is to analyze the association between EPA and DHA intake and all-cause and specific mortality in patients with diabetes.

## 2. Materials and methods

### 2.1. Study population

The National Health and Nutrition Examination Survey (NHANES) is a research program conducted by the National Center for Health Statistics of the Centers (NCHS) for Disease Control and Prevention (CDC) to assess the health and nutritional status of adults and children in the USA^1^. NHANES adopts a stratified multi-stage sampling design, which is nationally representative. The survey was conducted regularly until 1999, after which it became an ongoing program, and is released as a 2 years cycle. Details of the study design, sampling methods, and data collection have been described previously (20).

**FIGURE 1:**
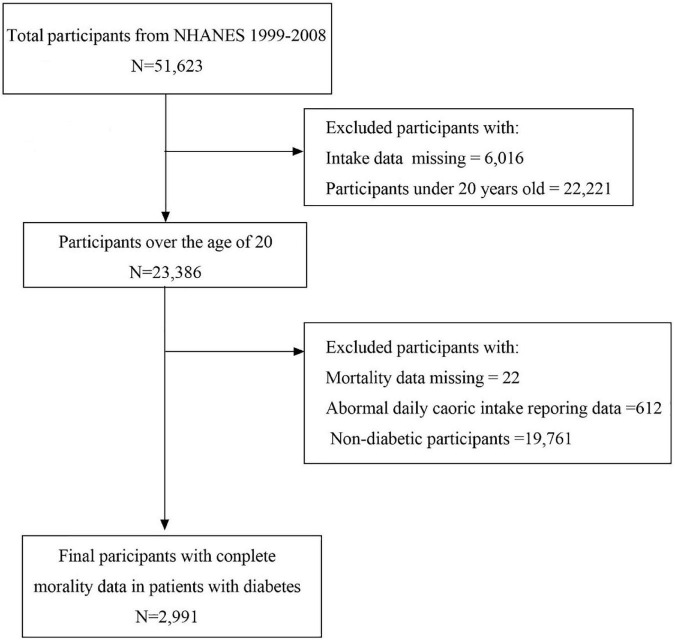
Flow chart of sample selection.

In this study, patients diagnosed with diabetes in the continuous NHANES database from 1999 to 2000 were selected and linked to the database from 2007 to 2008. The current analysis includes 51,623 participants from the NHANES (1999–2008) survey. The participants who were less than 20 years (*n* = 22,221) or non-diabetic (*n* = 19,761) and those with missing dietary intakes (*n* = 6016), abnormal calorie intake (male participants with calorie intake less than 800 or more than 8,000 and female participants with calorie intake less than 600 or more than 6,000, *n* = 612) (21), and missing mortality data (*n* = 22) were excluded from the analysis. Overall, 2,991 participants aged ≥20 from NHANES who were diagnosed with diabetes were included (Figure 1).

The CDC Research Ethics Review Committee approved the NHANES procedures and protocols and provided written informed consent to all participants. The analysis of this study only used publicly available data, the data involved were not directly obtained by participants, and ethical approval was not required.

### 2.2. Assessment of T2DM

Participants with at least one of the following conditions were identified as patients with T2DM: (1) fasting plasma glucose > 7.8 mmol/L, (2) taking diabetes drugs or insulin, and (3) HbA1c ≥ 6.5%.

### 2.3. Exposure measurement

Eicosapentaenoic acid (EPA) and docosahexaenoic acid (DHA) dietary intake was calculated by the United States Drug Administration’s Survey Nutrient Databases according to the 24 h dietary recall. The EPA intake in gm/day was divided into four categories (Q1 = 0, Q2 = 0.001–0.004, Q3 = 0.005–0.016, and Q4 ≥ 0.017), and DHA intake was categorized as ≤0.003, 0.004–0.028, 0.029–0.067, and >0.067.

### 2.4. Covariates measurement

Information on age, gender, ethnicity, education level, smoking status, alcoholic intake, nutrient intake, body mass index (BMI), and history of hypertension and dyslipidemia was collected using standardized questionnaires. Ethnicity was classified as non-Hispanic white, non-Hispanic black, Mexican American, and other. Smoking status was classified as never smoker, former smoker, and current smoker using two inquiries: “Have you smoked at least 100 cigarettes in your entire life?” and “Do you now smoke cigarettes?”. Current alcoholic intake was categorized as none (0 g/day), moderate drinking (0.1–27.9 g/day for men and 0.1–13.9 g/day for women), and heavy drinking (≥28 g/day for men and ≥14 g/day for women). Nutrient intake (e.g., total energy, carbohydrate, and protein intakes) was calculated by the United States Drug Administration’s Survey Nutrient Databases according to the 24 h dietary recalls. Hypertension was defined as being told by a doctor to have high blood pressure. Dyslipidemia was defined as HDL < 40 mg/dl or total cholesterol ≥ 200 mg/dl or low-density lipoprotein cholesterol (LDL-C) ≥ 130 mg/dl, or TG ≥ 130 mg/dl.

### 2.5. Assessments of outcome events

The primary outcome of this study was all-cause mortality assessed by the National Death Index. The underlying cause of death in secondary outcome was defined according to the international classification of diseases [9th edition (ICD-9) until 1998, and 10th edition (ICD-10) after that], including total CVD mortality (ICD I00-I09, I11, I13, I20-I51, and I60-I69), and CHD mortality (ICD I00-I09, I11, I13, and I20-I51).

### 2.6. Statistical analysis

Baseline characteristics were described across different levels of EPA and DHA intakes. We examined the demographic characteristics (age, gender, ethnicity, and education level), individual behavioral factors (smoking status, alcohol intake, and nutrient intake), and clinical risk factor distributions (BMI, serum C-reactive protein, and total cholesterol), as well as medical history characteristics (including prior hypertension and dyslipidemia) across the increasing quartiles of EPA and DHA intake. Differences among quartiles were tested by analysis of variance for continuous variables and χ^2^ test for categorical variables.

Cox proportional hazard regression models were used to estimate associations between EPA and DHA intakes and mortality risk (all-cause, CVD, and CHD). NHANES recommends using sample weights to calculate estimates that are representative of the U.S. civilian non-institutionalized population or any subpopulation of interest. “PROC SURVEYREG” was used in computing descriptive and regression analyses as this protocol account for both the weighted data as well as the complexity of sample design. The hazard ratios (HRs) and 95% confidence intervals (CIs) were calculated from the interview date to the date of death or censoring (31 December 2015), whichever came first. According to the previously selected covariates, model 1 was adjusted for age, model 2 added sex, ethnicity, education, BMI, alcohol, and smoking based on model 1, and model 3 added total energy intake, total carbohydrate intake, total protein intake, total dietary fiber intake, serum LDL-cholesterol, serum HDL-cholesterol, serum total cholesterol, and serum C-reactive protein (mg/dl) based on model 2. Model 4 added a history of hypertension and dyslipidemia on the basis of model 3. Missing values of continuous variables were replaced by mean values. In addition, stratified analyses were conducted for different age (age <60 and ≥60 years), sex (male and female), BMI (<25 and ≥25 kg/m^2^), and disease history (hypertension and dyslipidemia) groups to explore their effect modifications on the observed association by examining an interaction (cross-product) term of these dichotomous variables with the quartile variable of EPA and DHA intakes. All statistical analyses were performed based on SAS statistical software version 9.4 using a two-sided *P* < 0.05 as the significance level.

## 3. Results

### 3.1. Characteristics of participants

Among the 2,991 patients with diabetes aged ≥20, 1,652 (55.23%) were men. The mean age was 61.9 years, and more than half were over 60 years old (1882, 62.92%).

During 27,988 person-years (median follow-up of 9.1 years and maximum follow-up time was 16.8 years), a total of 1,091 participants died (of whom, 273 died of CVD, including 227 deaths from CHD). Participants with low intakes of EPA and DHA were more likely to be females, Mexican Americans, never drinkers, and hyperlipidemia, and hypertension patients than those with higher intakes, as shown in Table 1. People with high EPA intake also had higher serum total cholesterol, total energy intake, total carbohydrate, and total protein levels. People with high DHA intake had higher total energy and total protein intake.

**TABLE 1:** Baseline demographic characteristics of the study population, according to quartiles of eicosapentaenoic acid (EPA) and docosahexaenoic acid (DHA).

Characteristic	EPA (gm)					DHA (gm)				
	Q1 0	Q2 0.001–0.004	Q3 0.005–0.016	Q4 ≥0.017	*P*_*value*_	Q1 ≤0.003	Q2 0.004–0.028	Q3 0.029–0.067	Q4 >0.067	*P*_*value*_
Total N	871	586	783	751	–	777	726	747	741	–
Age, y	–	–	–	–	0.052	–	–	–	–	0.062
20–40	70 (11.7)	36 (7.7)	49 (9.3)	62 (10.8)	–	62 (11.0)	45 (8.3)	52 (10.3)	58 (10.7)	–
40-60	243 (38.8)	157 (35.7)	254 (45.2)	238 (41.7)	–	198 (33.8)	223 (43.1)	231 (41.5)	240 (44.6)	–
≥60	558 (49.6)	393 (56.7)	480 (45.5)	451 (47.5)	–	517 (55.2)	458 (48.6)	464 (48.2)	443 (44.7)	–
Gender	–	–	–	–	0.007	–	–	–	–	<0.001
Men	407 (45.3)	284 (47.7)	387 (51.1)	414 (53.6)		373 (46.6)	309 (44.7)	391 (51.4)	419 (55.3)	–
Women	464 (54.7)	302 (52.3)	396 (48.9)	337 (46.4)		404 (53.4)	417 (55.3)	356 (48.6)	322 (44.7)	–
Race/ethnicity^c^	–	–	–	–	<0.001	–	–	–	–	<0.001
Non-Hispanic white	222 (6.8)	147 (9.0)	215 (9.9)	146 (7.1)	–	177 (6.6)	176 (7.6)	219 (10.5)	158 (8.0)	–
Non-Hispanic black	84 (14.2)	48 (7.8)	67 (11.0)	89 (14.4)	–	72 (13.1)	62 (8.8)	69 (12.4)	85 (14.6)	–
Mexican American	360 (65.2)	251 (68.6)	299 (62.8)	261 (57.3)	–	72 (68.7)	62 (69.3)	69 (57.8)	85 (55.8)	–
Other	205 (13.8)	140 (14.6)	202 (16.2)	255 (21.2)	–	160 (11.6)	175 (14.3)	213 (19.3)	254 (21.6)	
Education	–	–	–	–	0.362	–	–	–	–	0.055
<High school	211 (12.3)	135 (14.8)	193 (13.0)	165 (13.2)	–	181 (12.4)	149 (10.6)	202 (16.4)	172 (13.6)	–
High school	395 (47.9)	266 (46.3)	326 (42.9)	322 (42.1)	–	353 (47.3)	325 (46.3)	320 (45.1)	311 (40.4)	–
>High school	264 (39.8)	184 (38.9)	262 (44.1)	264 (44.7)	–	242 (40.3)	251 (43.0)	223 (38.5)	258 (46.0)	–
BMI, kg/m^2^	–	–	–	–	0.823	–	–	–	–	0.413
<25.0 (normal)	154 (17.4)	101 (18.1)	140 (14.8)	140 (18.0)	–	148 (19.2)	115 (13.3)	141 (18.3)	131 (17.2)	–
25.0–29.9 (overweight)	274 (28.3)	184 (28.1)	229 (27.2)	212 (25.5)	–	244 (28.8)	226 (27.9)	220 (26.5)	209 (25.8)	–
≥30.0 (obese)	443 (54.3)	301 (53.8)	414 (58.0)	399 (56.5)	–	385 (52.0)	385 (58.8)	386 (55.2)	401 (57.0)	–
Alcohol	–	–	–	–	<0.001	–	–	–	–	–
Never drinker	764 (86.6)	512 (86.7)	665 (83.5)	611 (81.0)		690 (88.1)	630 (84.5)	630 (83.5)	602 (80.8)	0.001
Moderate drinking	60 (6.8)	28 (5.4)	65 (9.2)	83 (11.8)		41 (4.8)	49 (8.5)	67 (9.5)	79 (11.3)	
Heavy drinking	47 (6.6)	46 (7.9)	53 (7.3)	57 (7.2)		46 (7.1)	47 (7.0)	50 (6.9)	60 (7.9)	
Smoking	–	–	–	–	0.803	–	–	–	–	0.304
Never smoker	406 (46.9)	273 (46.2)	367 (48.5)	371 (51.3)	–	370 (46.4)	356 (49.0)	326 (46.5)	365 (51.5)	–
Former smoker	309 (32.7)	215 (36.2)	280 (35.1)	246 (30.1)	–	280 (34.4)	247 (34.5)	279 (34.2)	244 (29.9)	–
Current smoker	155 (20.4)	96 (17.6)	135 (16.4)	132 (18.6)	–	127 (19.2)	121 (16.5)	140 (19.3)	130 (18.6)	–
LDL-cholesterol (mg/dl)	108.96 ± 35.6	110.87 ± 39.36	109.7 ± 35.34	112.33 ± 39.34	0.094	105.98 ± 36.34	112.75 ± 38.64	110.61 ± 35.59	112.55 ± 38.62	0.363
HDL-cholesterol (mg/dl)	47.21 ± 13.75	48.61 ± 14.48	47.79 ± 14.09	47.85 ± 13.9	0.578	47.24 ± 13.87	47.92 ± 14.11	48.06 ± 13.99	48.02 ± 14.16	0.947
Total cholesterol (mg/dl)	198.48 ± 45.2	198.66 ± 47.3	197.53 ± 51.98	200.55 ± 51.93	<0.001	196.54 ± 46.69	200.73 ± 49.11	197.56 ± 49.35	200.51 ± 51.37	0.084
Serum C-reactive protein (mg/dl)	0.71 ± 1.42	0.72 ± 1.32	0.64 ± 0.93	0.64 ± 1.08	<0.001	0.67 ± 1.02	0.71 ± 1.58	0.63 ± 0.92	0.7 ± 1.21	< 0.001
Total energy intakes, kcal/day	1731.33 ± 767.53	1758.88 ± 775.59	1833.46 ± 772.77	2003.3 ± 878.64	<0.001	1696.42 ± 763.59	1755.35 ± 737.37	1833.29 ± 797.35	2046.95 ± 878.17	< 0.001
Total carbohydrate intakes, gm/day	221.88 ± 113.94	212.67 ± 100.28	217.72 ± 98.86	225.54 ± 105.58	<0.001	218.01 ± 106.62	219.03 ± 104.04	215.96 ± 105.78	226.74 ± 105.11	0.924
Total protein intakes, gm/day	65.93 ± 30.54	68.05 ± 33.34	74.24 ± 30.32	92.56 ± 42.7	<0.001	63.99 ± 31.04	67.12 ± 29.55	74.85 ± 30.11	95.25 ± 43.19	<0.001
Total dietary fiber intakes, gm/day	16.29 ± 10.78	15.08 ± 10	16.09 ± 9.43	15.84 ± 9.73	<0.001	16.3 ± 10.79	16.07 ± 10.09	15.36 ± 9.23	15.81 ± 9.92	<0.001
Dyslipidemia, %	701 (81.5)	467 (79.0)	621 (81.3)	597 (80.0)	0.937	617 (81.1)	591 (80.6)	590 (81.1)	588 (79.6)	0.652
History of hypertension, %	546 (59.1)	375 (64.9)	499 (61.5)	473 (62.0)	0.911	501 (62)	458 (63.8)	476 (59.1)	458 (61)	0.734

BMI, body mass index; CVD, cardiovascular disease; CHD, coronary heart disease.

### 3.2. DHA, EPA intake with Cardiovascular, and all-cause mortality

Table 2 shows the effects of EPA and DHA intake on all-cause mortality and death caused by cardiovascular diseases (including CHD) in patients with diabetes. EPA and DHA intakes were associated with a lower risk of mortality that reached the level of significance for CVD and CHD deaths but not all-cause deaths. The multivariable HRs (95% CIs) in a fully-adjusted model comparing participants in the highest vs. lowest quartiles of EPA intake were 0.55 (0.33–0.92; *P*-trend = 0.019) for CHD, 0.55 (0.36–0.83; *P*-trend = 0.005) for CVD, and 0.91 (0.70–1.18; *P*-trend = 0.264) for all-cause. The respective HRs comparing Q4 to Q1 of DHA were 0.60 (0.37–0.98; *P*-trend = 0.051) for CHD, 0.58 (0.38–0.89; *P*-trend = 0.014) for CVD, and 0.92 (0.72–1.18; *P*-trend = 0.481).

**TABLE 2:** Associations of the intake of eicosapentaenoic acid (EPA) and docosahexaenoic acid (DHA) with cardiovascular and all-cause mortality in United States adults aged at least 20 Years.

	EPA (gm)					DHA (gm)				
	Q1 0	Q2 0.001–0.004	Q3 0.005–0.016	Q4 ≥0.017	*P*_*trend*_	Q1 ≤0.003	Q2 0.004–0.028	Q3 0.029–0.067	Q4 >0.067	*P*_*trend*_
**CHD mortality**										
Deaths, no. (%)	86 (8.9)	44 (7.8)	57 (6.8)	40 (4.5)	0.007	77 (8.9)	58 (7.8)	50 (6.8)	42 (4.5)	0.012
Deaths/person-years	591/8436	280/5019	324/7315	261/7218	–	501/7116	388/6703	289/7091	279/7079	–
Unadjusted	1.00 [Reference]	1.09 (0.6, 1.98)	0.8 (0.56, 1.15)	0.51 (0.3, 0.86)	0.008	1.00 (Reference)	0.80 (0.51, 1.24)	0.58 (0.36, 0.94)	0.49 (0.30, 0.81)	0.014
Model 1	1.00 (Reference)	0.98 (0.55, 1.74)	0.88 (0.61, 1.26)	0.51 (0.31, 0.84)	0.007	1.00 (Reference)	0.84 (0.55, 1.29)	0.62 (0.39, 1.01)	0.54 (0.33, 0.87)	0.021
Model 2	1.00 (Reference)	0.97 (0.55, 1.71)	0.88 (0.62, 1.27)	0.51 (0.31, 0.82)	0.004	1.00 (Reference)	0.88 (0.57, 1.37)	0.63 (0.39, 1.03)	0.53 (0.33, 0.86)	0.013
Model 3	1.00 (Reference)	0.94 (0.54, 1.65)	0.88 (0.62, 1.25)	0.55 (0.33, 0.9)	0.014	1.00 (Reference)	0.90 (0.58, 1.37)	0.64 (0.39, 1.05)	0.58 (0.36, 0.95)	0.036
Model 4	1.00 (Reference)	0.91 (0.53, 1.58)	0.88 (0.62, 1.25)	0.55 (0.33, 0.92)	0.019	1.00 (Reference)	0.89 (0.58, 1.37)	0.66 (0.40, 1.08)	0.60 (0.37, 0.98)	0.051
**CVD mortality**										
Deaths, no. (%)	107 (10.8)	53 (9.7)	64 (7.9)	49 (5.3)	<0.001	97 (11.3)	68 (9.2)	60 (7.3)	48 (5.5)	<0.001
Deaths/person-years	753/8436	4682/5019	6942/7315	6916/7218	–	650/7116	453/6703	357/7091	305/7079	–
Unadjusted	1.00 (Reference)	1.11 (0.66, 1.86)	0.77 (0.54, 1.08)	0.50 (0.32, 0.77)	0.001	1.00 (Reference)	0.81 (0.55, 1.20)	0.61 (0.40, 0.95)	0.46 (0.30, 0.71)	0.002
Model 1	1.00 (Reference)	1.00 (0.61, 1.62)	0.84 (0.59, 1.19)	0.50 (0.33, 0.75)	<0.001	1.00 (Reference)	0.86 (0.59, 1.25)	0.65 (0.43, 0.99)	0.50 (0.33, 0.75)	0.002
Model 2	1.00 (Reference)	0.98 (0.60, 1.60)	0.84 (0.59, 1.19)	0.49 (0.33, 0.73)	<0.001	1.00 (Reference)	0.89 (0.60, 1.32)	0.67 (0.43, 1.04)	0.50 (0.33, 0.76)	0.002
Model 3	1.00 (Reference)	0.97 (0.59, 1.58)	0.85 (0.61, 1.20)	0.55 (0.36, 0.82)	0.003	1.00 (Reference)	0.91 (0.62, 1.35)	0.69 (0.44, 1.10)	0.56 (0.36, 0.87)	0.010
Model 4	1.00 (Reference)	0.94 (0.58, 1.52)	0.85 (0.61, 1.19)	0.55 (0.36, 0.83)	0.005	1.00 (Reference)	0.91 (0.62, 1.35)	0.71 (0.45, 1.11)	0.58 (0.38, 0.89)	0.014
**All-cause mortality**										
Deaths, no. (%)	347 (33.7)	216 (35.6)	282 (31.4)	246 (29.3)	0.031	313 (35.3)	267 (33.5)	266 (30.5)	245 (29.7)	0.031
Deaths/person-years	2313/8436	1297/5019	1749/7315	1590/7218	–	1970/7116	1703/6703	1738/7091	1536/7079	–
Unadjusted	1.00 (Reference)	1.27 (0.95, 1.68)	0.96 (0.76, 1.21)	0.87 (0.67, 1.13)	0.069	1.00 (Reference)	0.94 (0.78, 1.14)	0.82 (0.65, 1.03)	0.79 (0.63, 0.99)	0.057
Model 1	1.00 (Reference)	1.16 (0.89, 1.49)	1.03 (0.84, 1.26)	0.87 (0.68, 1.12)	0.087	1.00 (Reference)	0.99 (0.83, 1.16)	0.87 (0.69, 1.08)	0.85 (0.68, 1.06)	0.148
Model 2	1.00 (Reference)	1.13 (0.89, 1.45)	1.05 (0.85, 1.30)	0.89 (0.70, 1.15)	0.144	1.00 (Reference)	1.04 (0.86, 1.24)	0.89 (0.71, 1.11)	0.89 (0.70, 1.13)	0.249
Model 3	1.00 (Reference)	1.12 (0.88, 1.44)	1.05 (0.85, 1.30)	0.93 (0.72, 1.20)	0.298	1.00 (Reference)	1.03 (0.86, 1.23)	0.89 (0.70, 1.12)	0.93 (0.73, 1.18)	0.468
Model 4	1.00 (Reference)	1.09 (0.86, 1.39)	1.04 (0.84, 1.29)	0.91 (0.70, 1.18)	0.264	1.00 (Reference)	1.02 (0.85, 1.22)	0.89 (0.71, 1.13)	0.92 (0.72, 1.18)	0.481

Values are *n* or weighted hazard ratio (95% confidence interval). Model 1, adjusted for age. Model 2, model 1 +sex, race/ethnicity, education, BMI, alcohol, and smoking. Model 3, model 2 +totals energy intake, total carbohydrate intake, total protein intake, total dietary fiber intakes, serum LDL-cholesterol, serum HDL-cholesterol, serum total cholesterol, and serum C-reactive protein (mg/dl). Model 4, model 3 +history of hypertension and dyslipidemia. CVD, cardiovascular disease; CHD, coronary heart disease; BMI, indicates body mass index.

### 3.3. DHA, EPA intake with cardiovascular, and all-cause mortality among subpopulations

**FIGURE 2:**
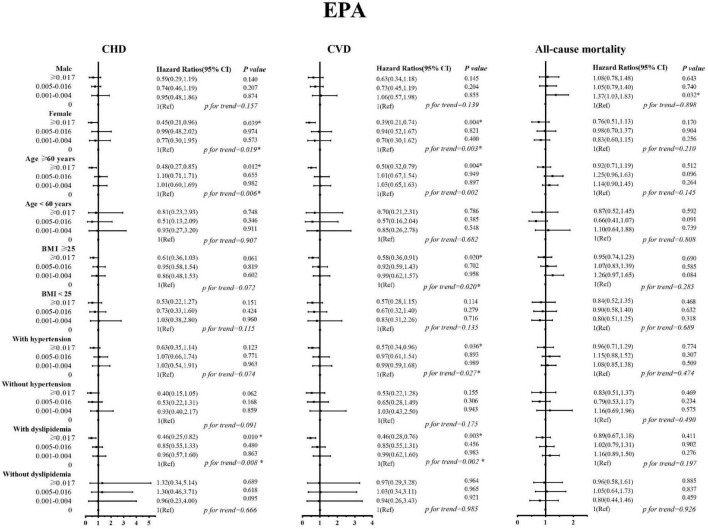
Stratified analyses by sex, age, body mass index, history of hypertension, and history of dyslipidemia for the associations between EPA and the mortality from cardiovascular disease, coronary heart disease, and all-causes.

**FIGURE 3:**
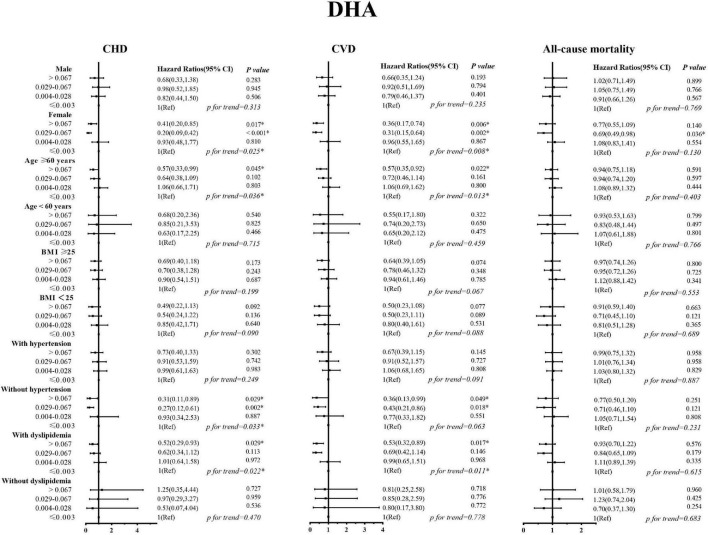
Stratified analyses by sex, age, body mass index, history of hypertension, and history of dyslipidemia for the associations between DHA and the mortality from cardiovascular disease, coronary heart disease, and all-causes.

Sex, age, BMI, and history of hypertension and dyslipidemia modified the associations of EPA and DHA with CVD (including CHD) mortality (Figures 2, 3). The inverse association with higher EPA intake for CVD was evident in patients who are old, females, those with higher BMI, have a history of hypertension, and in patients with dyslipidemia; the *P-*values were 0.004, 0.004, 0.020, 0.036, and 0.003, respectively. The inverse association with higher DHA intake for CVD was evident in patients who are old, females, those with higher BMI, have a history of hypertension, and in patients without dyslipidemia; the *P-*values were 0.022, 0.0.006, 0.049, and 0.017, respectively. The inverse trend was seen among those who are old, females, and dyslipidemia patients with CVD, but did not reach the level of significance in both strata; the *P*-interaction with sex, age, BMI, and history of hypertension and dyslipidemia were ≥ 0.05 for EPA and DHA.

## 4. Discussion

In this nationally representative prospective study, we found that higher intakes of DHA and EPA were inversely associated with the mortality risk, especially that of CVD and CHD in patients with diabetes. The association existed after controlling for demographics, behavioral, and lifestyle factors. At the same time, in subgroup analysis, we found that the association trends of EPA and DHA with the reduced death risk remained robust among patients with diabetes, especially among those who are old, females, those with higher BMI, and patients with dyslipidemia.

High-dose EPA and DHA have independent effects on chronic inflammation through downstream lipid mediators, which can reduce blood viscosity and enhance vascular elasticity, thus reducing the risk of poor prognosis of myocardial infarction (MI) and heart failure (22). Also, studies have shown that EPA and DHA can regulate cytokine expression to affect the inflammatory response of peripheral blood monocytes (22), have a protective effect on the heart, inhibit inflammation, and improve clinical outcomes (23). The supplementation of high-dose EPA and DHA for patients with CVD could improve their left ventricular function and reduce the incidence and recurrence risk of cardiovascular events (24). A review study reported that long-term consumption of ω-3 fatty acids was associated with about a 40% reduction in adult mortality and incidence rate from cardiovascular diseases, such as MI and arrhythmia (25). Our study also demonstrated that EPA and DHA intakes were associated with CVD and CHD mortality (*P* trend were 0.005 and 0.014, respectively).

Epidemiological studies have shown that lifestyle interventions, including dietary interventions, play a very important role in the progression, prevention, and management of diabetes (26, 27). The International Diabetes Federation (IDF) also made it clear that a healthy lifestyle is the cornerstone of type 2 diabetes treatment. Strengthening lifestyle intervention can not only prevent the occurrence of chronic diseases such as diabetes but also reduce the risk of diabetes-related macrovascular and microvascular complications. Dietary control ranks first among the five principles of modern comprehensive treatment. However, analyses have shown that diet improvement such as increasing omega-3, omega-6, or total PUFA has little or no effect on fasting blood glucose, glycated hemoglobin, or insulin resistance in patients with diabetes (28). Our study showed that the intake of EPA and DHA, especially over 0.017 of EPA and over 0.067 of DHA, had a certain protective effect on the death outcome of patients with diabetes, confirming the beneficial outcomes of LC-PUFAs on diabetes mellitus.

Patients with diabetes often suffer from oxidative stress injury and disturbance of glucose and lipid metabolism disorders. LC-PUFAs can not only regulate lipid metabolism in DMs but also improve insulin sensitivity and visceral obesity. Insulin resistance is one of the main pathogenesis of diabetes, which is mediated by a variety of inflammatory cells and inflammatory factors. Studies have shown that EPA and DHA, such as deep-sea fish oil, can improve insulin resistance and decrease fasting serum insulin levels to some extent in patients with diabetes (29). In addition, LC-PUFAs can promote the secretion of adiponectin, promote fat oxidation, and further increase insulin sensitivity. This may also explain our results to a certain extent, indicating that immune nutritional support can be used for nutritional therapy in patients with diabetes.

Diabetes is one of the important risk factors for CVD. Epidemiological evidence has shown that the presence of abnormal blood glucose or diabetes is associated with increased morbidity and mortality from vascular disease. Some studies have shown that there may be gender differences (30). However, the cause of gender differences in microvascular complications in diabetic populations remains uncertain. Lee et al. (31) have reported that the all-cause mortality of women with diabetes is similar to or higher than that of patients with previous CVD (HR = 1.25), and the risk of death is higher than that of men. But Isasi et al. (32) reported that sex hormones can protect women before menopause and reduce the CVD risk in women, unlike men. In developed industrialized countries, the incidence of CVD is nearly three to four times higher in men than that in women. The existence of underlying diabetes causes women to lose this protective effect.

We explored possible gender differences in the intake of LC-PUFAs for cardiovascular and all-cause mortality in patients with diabetes. The results showed that the intake of EPA and DHA at different levels was negatively correlated with the risk of cardiovascular events in patients with diabetes. For the occurrence of cardiovascular and all-cause mortality, women appear to be benefited more than men from consuming high levels of LC-PUFA. In women with diabetes, a high intake of EPA (≥0.017) reduced the risk of adverse clinical outcomes, especially CVD and CHD mortality (HR = 0.39 and 0.45, *P* < 0.05). The same conclusion was also found in DHA (>0.067), the risk of death from CVD and CHD was reduced by 0.64 and 0.59. However, we found that a similar protective effect was not statistically significant in men with diabetes. Overall, this study suggests that an appropriate level of nutrients can alleviate the risk of adverse clinical outcomes to a certain extent in patients with diabetes, and the protective effect is more pronounced for female patients.

Evidence from cohort studies suggested that physical activity and weight loss are associated with a reduced risk of macrovascular complications (33, 34). This study shows that compared with patients with a BMI <25 kg/m^2^, the protective effect of nutrient intake on adverse clinical outcomes in overweight patients is more obvious, and a certain dose-related trend can be seen. That is, with the increase in EPA and DHA intake, the protective effect on the risk of CVD and CHD mortality is enhanced. Although this protective effect did not appear to be statistically significant in our study, the trend in the results also suggests that dietary modifications, such as high-dose intake of EPA and DHA in patients with diabetes who have high levels of BMI (≥25.0 kg/m^2^), may have a beneficial effect.

In dyslipidemia stratified analysis, a significant trend of EPA and DHA intakes on history with dyslipidemia and CVD and CHD mortality was found. Higher EPA and DHA intakes significantly reduced the risk of CVD and CHD mortality and may provide better protection in dyslipidemia people. Patients with diabetes usually experience increased coagulation factor VIII and increased platelet function, which will further accelerate the formation of atherosclerotic thrombosis and cause arterial lumen occlusion (35). EPA and DHA can regulate the lipid metabolism process by increasing fatty acid β-oxidation, reducing the activity of liver enzymes that promote triacylglycerol synthesis, reducing serum TG levels, and increasing the level of HDL (36, 37). Several studies have found that higher levels of long-chain ω-3 PUFAs intakes could effectively prevent the onset of recent MI, reduce triacylglycerol levels, maintain myocardial function, and especially reduce the risk of major coronary events in patients with hypercholesterolemia (37–39). In stratified analysis, we found that, in diabetic patients with dyslipidemia, The HRs of the highest dose EPA and DHA were 0.46 and 0.53 for CVD, and 0.46 and 0.52 for CHD, respectively.

### 4.1. Strengths and limitations

Major strengths of the study include screening diabetics samples from a large sample of the general population with racial/ethnic and geographic diversity from the NHANES survey. Nutritional intake assessment based on the United States Drug Administration’s Survey Nutrient Databases was more accurate according to the 24 h dietary recall, and we weighted the hazard ratios to make the results more reliable. However, the study has some limitations. First, we cannot avoid possible measurement errors based on the relevant self-reported information. Second, we did not capture the changes in the lifestyle of the population in different periods, so repeated measurement studies can be considered in the future. Third, although we adjusted the comorbidity of patients at baseline, key personal characteristics, and conducted a sensitivity analysis to rule out major chronic disease, residual confounding still cannot be eliminated completely.

## Conclusion

In summary, higher levels of EPA and DHA intake were inversely associated with the risk of CVD and CHD mortality in U.S. adults with diabetes. The inverse trend was seen in patients who are old, females, those with higher BMI, have a history of dyslipidemia, and in diabetic patients with CVD.

## Data availability statement

The original contributions presented in this study are included in the article/supplementary material, further inquiries can be directed to the corresponding author.

## Author contributions

JC supervised the study. JC, NH, and FW designed the study. NH, XZ, SL, WM, KL, HS, and EE collected and organized the data. NH analyzed the data. JC, NH, FW, and EE interpreted the results. FW wrote the first draft. All authors read and approved the final manuscript.

## Footnotes

1
https://www.cdc.gov/nchs/nhanes/index.htm
